# High prevalence of Berardinelli-Seip Congenital Lipodystrophy in Rio Grande do Norte State, Northeast Brazil

**DOI:** 10.1186/s13098-017-0280-7

**Published:** 2017-10-13

**Authors:** Lázaro Batista de Azevedo Medeiros, Verônica Kristina Cândido Dantas, Aquiles Sales Craveiro Sarmento, Lucymara Fassarella Agnez-Lima, Adriana Lúcia Meireles, Thaiza Teixeira Xavier Nobre, Josivan Gomes de Lima, Julliane Tamara Araújo de Melo Campos

**Affiliations:** 1 Faculdade de Ciências da Saúde do Trairi, Universidade Federal do Rio Grande do Norte, Santa Cruz, RN Brazil; 2 Laboratório de Biologia Molecular e Genômica, Departamento de Biologia Celular e Genética, Centro de Biociências, Universidade Federal do Rio Grande do Norte, Natal, RN Brazil; 3 Departamento de Nutrição Clínica e Social, Escola de Nutrição, Universidade Federal de Ouro Preto, Ouro Preto, MG Brazil; 4 Departamento de Medicina Clínica, Hospital Universitário Onofre Lopes (HUOL)/UFRN, Natal, RN Brazil

**Keywords:** BSCL, High prevalence, Brazil, RN

## Abstract

**Background:**

Berardinelli-Seip Congenital Lipodystrophy (BSCL) is a rare disease characterized by the almost complete absence of adipose tissue. Although a large number of BSCL cases was previously identified in Rio Grande do Norte (RN), a state in Northeast Brazil, its prevalence in RN regions and municipalities remains unknown. The purpose of this study was to better characterize the prevalence of BSCL in RN.

**Methods:**

A descriptive study was conducted using secondary data obtained from the Association of Parents and People with BSCL of RN to determine its prevalence. The patients’ socio-demographic characteristics and geolocalization were analyzed.

**Results:**

We estimated a total of 103 BSCL cases in RN, resulting in a prevalence of 3.23 per 100,000 people. The Central Potiguar mesoregion, Seridó territory, Carnaúba dos Dantas and Timbaúba dos Batistas municipalities had a much higher prevalence of BSCL, with 20.56, 20.66, 498.05 and 217.85 per 100,000 people, respectively.

**Conclusions:**

Together, our results showed that BSCL is highly prevalent in RN and confirmed that our state has one of the highest prevalences of this lipodystrophy worldwide. More studies are still needed to better estimate the prevalence and incidence of BSCL in RN as well as in other states in Brazil.

*Trial registration* Study Number 31809314.0.0000.5568

## Background

Berardinelli-Seip Congenital Lipodystrophy (BSCL) is a rare autosomal recessive disease characterized by a near-complete absence of adipose tissue from birth [1]. This lipodystrophy is associated with fatty liver, hypertriglyceridemia, hyperinsulinemia, type 2 diabetes, acanthosis nigricans, prominent musculature and other clinical conditions [2–5]. It was initially described in 1954 by the Brazilian doctor Waldemar Berardinelli [1] and in 1959 by the Norwegian doctor Martin Seip [6].

There are two major subtypes of BSCL: BSCL1 and BSCL2. The molecular cause of the first subtype is a mutation in a gene encoding 1-acylglycerol-3-phosphate *O*-acyltransferase 2 (1-AGPAT 2), an enzyme expressed predominantly in adipose tissue that is involved in the biosynthesis of triacylglycerols and phospholipids [7, 8]. In contrast, BSCL2 is caused by a mutation in a gene encoding seipin, an endoplasmic reticulum transmembrane protein that is involved in adipogenesis and lipid droplet formation [9, 10]. Although seipin is mainly localized to adipose tissue, it also has functional roles in the testes and brain [9–11].

The prevalences of BSCL in different countries have been established in the medical literature and databanks specializing in rare genetic syndromes [3, 12, 13]. Although its prevalence worldwide is estimated to be 1 in 12 million people [12], there is some variation among individuals of different ethnicities. In the US and Norway, the prevalences of this disease are 1 in 10 million and 1 in 1 million people, respectively [3, 13]. However, in Lebanon, Portugal and Oman, its prevalences are 1 in 200,000, 1 in 500,000 and 1 in 25,000 people, respectively [13].

Although the first BSCL case was reported in Brazil, its prevalence in this country has not yet been investigated. Limited information is available about the prevalence of BSCL in some regions of Brazil. Previous reports have indicated that cases of this disease have occurred in two regions of Brazil: Rio Grande do Norte (RN) state, located in the northeastern region, and Minas Gerais (MG) state, located in the southeastern region [14, 15]. Clinical and laboratory data of BSCL patients from RN have recently been reported by Lima and co-workers, revealing that a specific mutation in the BSCL2 gene is predominant in BSCL patients from this state [16]. Although several cases of BSCL have been reported in RN, there is no information concerning the distribution of cases among the regions and municipalities of RN and Brazil. These data could help establish that Brazil has one of the highest prevalences of BSCL worldwide.

The aim of this study was to estimate the prevalence of BSCL in RN, Brazil, and therefore to provide appropriate epidemiologic data in order to help the Brazilian Health System promote the correct multidisciplinary management of BSCL patients, focusing on genetic counseling for families, prenatal diagnosis and the screening of family members, as well as to improve future studies on this lipodystrophy in Northeast Brazil.

## Methods

### Study design and data collection

A descriptive study was performed using data obtained from the Association of Parents and People with BSCL of RN (*Associação dos Pais e Pessoas com a Síndrome de Berardinelli do Estado do Rio Grande do Norte*—*ASPOSBERN*), located in the Currais Novos municipality of RN. It is a non-profit organization founded in 1998 that aims to improve the quality of life of BSCL patients and their families. The analysis was performed after data collection for all BSCL patients (living and dead) who were assisted by *ASPOSBERN* between 1998 and March 2015. It is important to note that the ASPOSBERN Association plays an important role in the management of BSCL patients diagnosed by qualified physicians that aid this Association. A map of the RN municipalities with reported BSCL cases was constructed using the TabWin program (DATASUS/Ministério da Saúde).

### Genotyping

29 BSCL patients with genetic diagnosis were classified as BSCL1 or BSCL2 on the basis of mutational analysis. All BSCL1 patients and almost all BSCL2 patients were genotyped by Lima et al. [16]. The genotyping for the new BSCL2 patients that were included in our analysis was performed according to Lima et al. [16]. Briefly, Genomic DNA was extracted from peripheral blood cells using Illustra triplePrep Kit (GE Healthcare). 325dupA variant in *BSCL2* gene (rs786205071) was evaluated by PCR–RFLP using the primers TGGCAACATGATGGTTCACT (Forward) and CCATTCTGATCCTGCCATCT (Reverse). Then, the amplicon was submitted to digestion by 1U of HpaI enzyme (New England BioLabs) for 3 h at 37 °C. The presence of the 325dupA variant produce two fragments of 328 and 172 bp after digestion. The fragments were stained with SYBR^®^ Green I Nucleic Acid Stain (Lonza) and separated in a 2% agarose gel (GE Healthcare). The amplification products were visualized under Chemi-Doc equipment (Bio-Rad).

### Statistical analysis

The variables assessed in this study included age, sex, level of education and city of residence. The data were plotted using Excel software (version 2010). The numbers of BSCL cases by municipality were expressed as absolute numbers. The prevalence was calculated by dividing the total number of cases registered by *ASPOSBERN* from 1998 to March 2015 by the resident population of the municipality, mesoregion or state, as determined from 2010 census data reported by the *Instituto Brasileiro de Geografia e Estatística* (*IBGE*) [17]. For the RN territories, the prevalence was calculated by dividing the total number of *ASPOSBERN* cases by the population reported by the *Sistema de Informações Territoriais* (*SIT*) [18].

## Results

### Socio-demographic characteristics of BSCL patients

Of the 103 BSCL cases registered by *ASPOSBERN*, 59 and 44 were diagnosed before and after its establishment in 1998, respectively. Since the large number of BSCL patients in RN is due to consanguineous marriage, and we observed an increased incidence of diagnosed BSCL patients, it was easier to detect new BSCL cases in our county. At the end of the 20th century, 74 patients (72%) were evaluated personally by qualified physicians due to the absence of genetic tests to detect lipodystrophic patients in RN. Diagnosis was based on a history and physical examination. Only 29 patients (28%) were diagnosed with BSCL by qualified physicians and genetic tests, which were performed by Lima et al. [16].

Although some data were not available for patients registered before 1998, we found that 62.8% of our patients were female and that only 36.0% (n = 37) were still alive. In addition, between 1998 and 2015, *ASPOSBERN* collected data for each BSCL patient, including their sex, age, level of education and vital signs, among whom 70.4% were alive (n = 31), and 56.8% were female (n = 25); the mean age was 23.2 ± 16.7 years old. According to the education data, 47.7% (n = 21) of the BSCL patients did not complete elementary school, 11.4% (n = 5) completed elementary school, 6.8% (n = 3) completed a high level of education, and education information was not available for 34.1% (n = 15). Among the patients who were alive (n = 31), the mean age was 26.4 ± 15.9 years. Moreover, the minimum and maximum ages were 1 and 57 years, respectively (Table 1).

**Table 1:** Characteristics of BSCL patients from the state of Rio Grande do Norte between 1998 and 2015

Characteristics of BSCL patients from RN^a^	N (%)
Survival	44 (100)
Alive	31 (70.4)
Dead	13 (29.6)
Age group (years)	44 (100)
Children (up to 11)	8 (18.2)
Adolescents (12–18)	3 (6.8)
Adults (19–59)	19 (43.2)
Unknown	14 (31.8)
Sex	44 (100)
Female	25 (56.8)
Male	19 (43.2)
Education level	44 (100)
Did not complete elementary school	21 (47.7)
Completed elementary school	5 (11.4)
Completed a higher level of education	3 (6.8)
Unknown	15 (34.1)

*N* absolute number of cases

^a^For this analysis, only data for BSCL patients assisted by *ASPOSBERN* between 1998 and March 2015 were included

### Geolocalization

From the total number of cases, we estimated that the prevalence of BSCL was 3.23 in 100,000 inhabitants of RN (Table 2). Furthermore, the distributions of BSCL cases in RN by mesoregion, territory and municipality are presented in Tables 2, 3 and 4, respectively. First, we established the prevalences of BSCL in all RN mesoregions. Central Potiguar had the highest number of BSCL cases in RN, with a prevalence of 20.56 in 100,000 inhabitants (95% CI 15.97–25.16) (Table 2).

**Table 2:** BSCL prevalences in the Rio Grande do Norte state mesoregions

Geographic area^a^	N^b^	P	95% CI
Rio Grande do Norte	103	3.23	(2.60 to 3.85)
Oeste Potiguar^a^	10	1.13	(0.43 to 1.83)
Central Potiguar^a^	77	20.56	(15.97 to 25.16)
Agreste Potiguar^a^	1	0.24	(− 0.23 to 0.71)
Leste Potiguar^a^	15	1.01	(0.50 to 1.53)

*N* absolute number of cases, *P* prevalence per 100,000 inhabitants, *CI* confidence interval

^a^According to IBGE, 2016

^b^According to ASPOSBERN, March 2015

**Table 3:** BSCL prevalences in the Rio Grande do Norte state territories

Territories^a^	N^b^	P	95% CI
Alto Oeste	4	2.04	(0.04 to 4.03)
Mato Grande	1	0.45	(− 0.4 to 1.32)
Seridó	77	26.04	(20.22 to 31.85)
Sertão do Apodi	5	2.99	(0.36 to 5.60)
Terras Potiguares	15	1.26	(0.62 to 1.90)
Trairi	1	0.75	(− 0.72 to 2.22)

*N* absolute number of cases, *P* prevalence per 100,000 inhabitants

^a^According to SIT, 2016

^b^According to *ASPOSBERN*, March 2015

**Table 4:** BSCL prevalences in the Rio Grande do Norte state municipalities

Municipality	RN mesoregion^a^	RN territory	N^b^	P	95% CI
Caicó	Central Potiguar	Seridó	5	7.97	(0.98–14.96)
Carnaúba dos Dantas	Central Potiguar	Seridó	37	498.05	(337.96–658.13)
Currais Novos	Central Potiguar	Seridó	5	11.72	(1.44–21.99)
Jardim de Piranhas	Central Potiguar	Seridó	5	37.02	(4.57–69.46)
Jardim do Seridó	Central Potiguar	Seridó	5	41.28	(5.10–77.45)
Natal	Leste Potiguar	Terras Potiguares	12	1.49	(0.64–2.33)
Timbaúba dos Batistas	Central Potiguar	Seridó	5	217.85	(27.10–408.62)

*N* absolute number of cases, *P* prevalence per 100,000 inhabitants

^a^According to IBGE census data, 2010

^b^According to *ASPOSBERN*, March 2015

To generate a more precise map of the main RN territories with BSCL cases, we also determined the prevalences in Alto Oeste, Mato Grande, Seridó, Sertão do Apodi, Terras Potiguares and Trairi. The analysis revealed that the prevalences of BSCL in all territories were higher than the worldwide prevalence (1 in 12,000,000). However, Seridó and Sertão do Apodi had the greatest prevalences, at 26.04 (95% CI 20.22–31.85) and 2.99 (95% CI 0.36–5.60) per 100,000 inhabitants, respectively (Table 3).

For the analysis of BSCL cases according to the RN municipalities, we included only municipalities with more than 5 reported cases. The results revealed that Carnaúba dos Dantas and Timbaúba dos Batistas, which are both in the Seridó region, had the highest prevalences of BSCL in RN (Table 4). Currais Novos city, where *ASPOSBERN* is located, had a BSCL prevalence of 11.72 in 100,000 people. Additional municipalities in RN, such as Acari, Apodi, Jaçanã, Messias Targino, and Parnamirim, in addition to others, had too few cases to allow for an estimation of the prevalence.

Our data also demonstrated that a lower prevalence of BSCL was found in urban areas (15%), which included the Terras Potiguares territory, compared with rural areas (85%), which included the other territories.

**Fig. 1:**
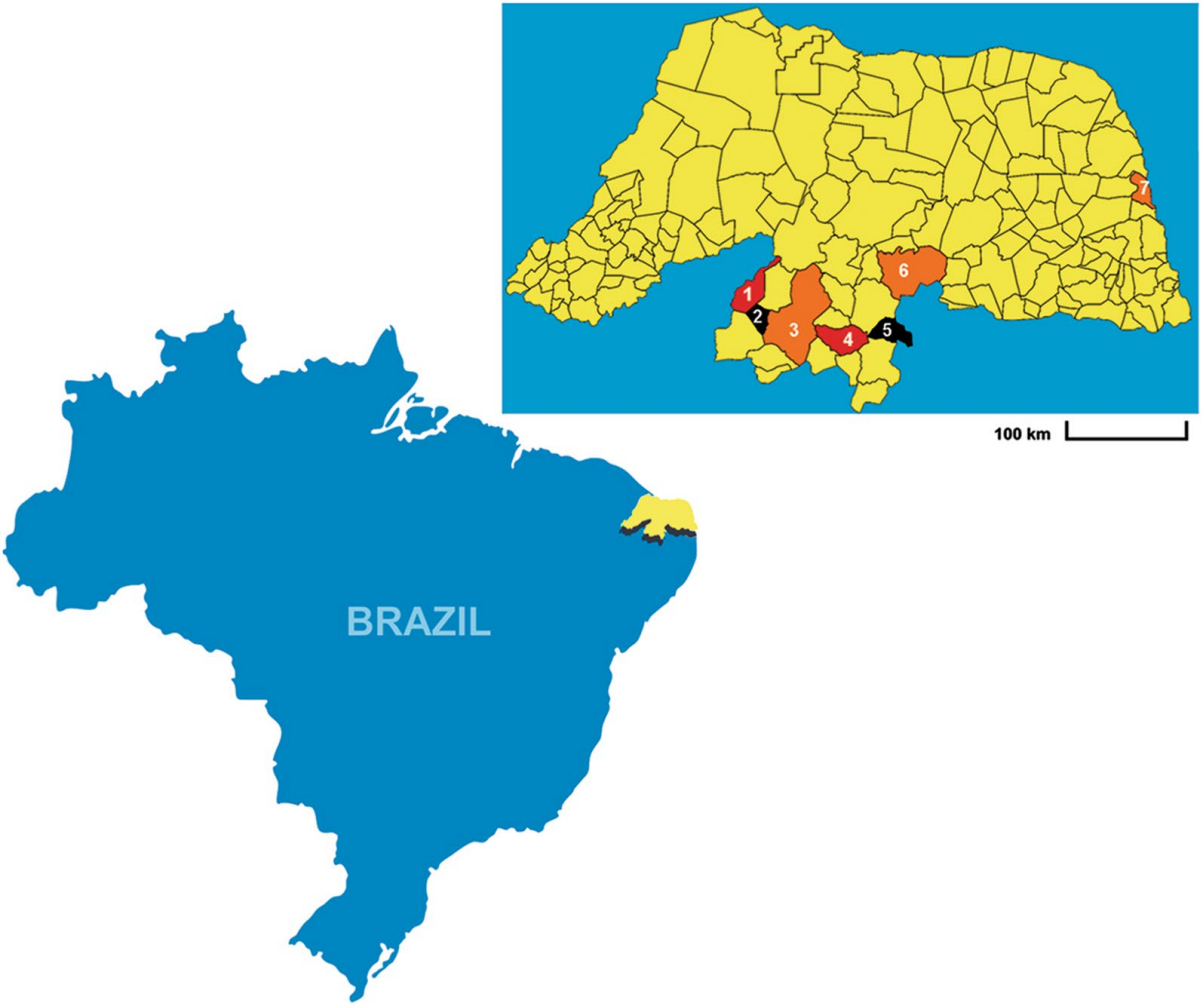
Map of the Rio Grande do Norte state showing the municipalities where BSCL cases were reported. The prevalence rates are shown in different colors: < 20/100,000 (orange); 20–40/100,000 (red); and > 40/100,000 (dark gray). 1: Jardim de Piranhas; 2: Timbaúba dos Batistas; 3: Caicó; 4: Jardim do Seridó; 5: Carnaúba dos Dantas; 6: Currais Novos; 7: Natal. The map was constructed using the TabWin Program (DATASUS/Ministério da Saúde)

To provide a better overview of the BSCL case distribution in RN, we constructed a map to distinguish the RN municipalities according to the prevalence per 100,000 inhabitants. Notably, the Central Potiguar mesoregion contained many municipalities with high BSCL prevalences (20–40/100,000 and > 40/100,000), whereas the municipalities in the other RN mesoregions mainly had prevalences of < 20/100,000 (Fig. 1).

## Discussion

Our investigation revealed a high prevalence of BSCL (3.23 in 100,000 inhabitants) in the state of RN, Northeast Brazil. This lipodystrophy affects individuals of all ethnicities, and its worldwide prevalence is estimated to be 1 in 12 million people [3]. The BSCL prevalences in many countries, such as the US, Norway, Lebanon, Portugal and Oman, have been previously reported [3, 13]. Cases of this type of congenital lipodystrophy have also been reported in China and Japan [19, 20].

Our study of BSCL prevalence in RN showed that the Central Potiguar mesoregion had a significantly high prevalence. Unsurprisingly, the Seridó territory, where BSCL cases have been reported in 12 municipalities, is located in the Central Potiguar mesoregion (Table 3). Because Brazil is a developing country with a large area and great socioeconomic and cultural diversity, the regional differences in RN may reveal the specific epidemiological characteristics of BSCL patients.

A preliminary study performed by a mother of a BSCL patient from the Currais Novos municipality showed that consanguineous marriages, which mainly occur in municipalities of the Seridó territory, are responsible for the elevated prevalence of BSCL in RN. This finding has been confirmed by Lima et al. [16], who verified the frequent occurrence of consanguineous marriages in Seridó, a RN territory where new BSCL cases are diagnosed every year. Thus, a map showing the distribution of BSCL cases among the municipalities of RN can be used to help the government detect new cases of this lipodystrophy, which would allow for the prediction of the health requirements of these patients and the prioritization of the provision of genetic services to affected families from RN as well as those from other Brazilian states.

Although *ASPOBERN* did not report the education level of all BSCL patients assisted from 1998 to 2015, we found that several BSCL patients in our study had a low education level. Twenty-one patients (47.7%) had not completed elementary school, 5 patients (11.4%) had completed elementary school, and 3 patients (6.8%) had a higher level of education. According to data obtained from *Instituto Brasileiro de Geografia e Estatística (IBGE),* these finding are similar to the socio-demographic data from the northeast population, which showed that 47.6% of Northeast people had not completed elementary school, 9.5% completed elementary school, and 4% had a higher level of education [21].

Data from the literature indicate that BSCL2 patients have an increased risk of cardiomyopathy and intellectual impairment compared with BSCL1 patients [22, 23], suggesting that seipin, the protein encoded by the *BSCL2* gene, is involved in the regulation of neuronal functions. Lima et al. have reported that most BSCL patients from RN have a BSCL2 gene mutation, which may be related to the increased incidence of premature death among these patients [16]. Together, our data concerning the educational levels indicated that the occurrence of the BSCL2 mutation found in RN may not be related to the intellectual profiles of these patients.

Importantly, we analyzed only cases registered by *ASPOSBERN*, and thus, we may have underestimated the prevalence of BSCL in RN. Another limitation of this study is that we analyzed secondary data; therefore, many important characteristics necessary for evaluating the real prevalence were not collected.

This is the first report of the prevalence of BSCL in Brazil. Because BSCL cases have also been reported in other Brazilian states [15], the BSCL prevalence that we have reported is an underestimation. The government of the state of RN does not currently report epidemiologic data on BSCL; only *ASPOSBERN* has attempted to obtain these data. The potential reasons for the lack of Brazilian BSCL studies are as follows: (i) the large physical size of Brazil; (ii) the absence of a healthcare system that could diagnose BSCL in children; (iii) Brazilian health professionals’ lack of knowledge on BSCL; and (iv) the low number of studies conducted in Brazil concerning Berardinelli-Seip syndrome. Research on BSCL in Brazil is limited compared with that in European countries and the US [2–7, 9, 10, 12, 13, 20, 22, 23].

Our data have indicated that the prevalence of BSCL is high in the state of RN, Brazil. Considering the wide regional diversity and the increasing number of BSCL cases in Brazil, more studies are needed to improve the understanding of its epidemiological characteristics as well as the quality of care provided by clinicians to BSCL patients. Thus, annual reporting of the BSCL prevalence in Brazil is essential for improving the care of BSCL patients, the knowledge of health professionals concerning this type of lipodystrophy, and research on BSCL. Therefore, it is very important to implement an effective system for reporting the prevalence of BSCL in Brazil, which would ultimately help provide direct resources to these patients.

## Conclusions

Several cases of BSCL have been reported in RN, but there is no information about the distribution of BSCL among the regions and municipalities of Brazil. Here, we have shown that the BSCL prevalence is high in RN (3.23 per 100,000 people) with emphasis on the Central Potiguar mesoregion, Seridó territory, and Carnaúba dos Dantas and Timbaúba dos Batistas municipalities. Furthermore, most of the patients assisted by ASPOSBERN between 1998 and March 2015 were female adults who had not completed elementary school. The high prevalence of this syndrome highlights the urgent requirement to develop efforts directed at monitoring the incidence of BSCL in Northeast Brazil with a focus on genetic counseling for families. Additionally, more studies are required to better estimate the prevalence and incidence of this condition in additional states of Brazil, which may have important implications for the health care sector.

## Abbreviations

AGPAT: acylglycerol-3-phosphate *O*-acyltransferase
ASPOSBERN: Association of Parents and People with Berardinelli Syndrome of *Rio Grande do Norte* (*Associação dos Pais e Pessoas com a Síndrome de Berardinelli do Estado do Rio Grande do Norte*)
BSCL: Berardinelli-Seip Congenital Lipodystrophy
CI: confidence interval
FACISA: *Faculdade de Ciências da Saúde do Trairi*
IBGE: *Instituto Brasileiro de Geografia e Estatística*
MG: *Minas Gerais*
RN: *Rio Grande do Norte*
SIT: *Sistema de Informações Territoriais*
UFRN: Federal University of *Rio Grande do Norte* (*Universidade Federal do Rio Grande do Norte*)
US: United States
